# Hereditary breast and ovarian cancer (HBOC): review of its molecular characteristics, screening, treatment, and prognosis

**DOI:** 10.1007/s12282-020-01148-2

**Published:** 2020-08-29

**Authors:** Reiko Yoshida

**Affiliations:** Showa University Advanced Cancer Translational Research Institute, 1-5-8 Hatanodai, Shinagawa-ku, Tokyo, 142-8555 Japan

**Keywords:** Hereditary breast and ovarian cancer, BRCA, *BRCA1/2* mutations, HBOC

## Abstract

Breast cancer is a common cancer affecting a large number of patients. Notably, 5–10% of all breast cancer patients are genetically predisposed to cancers. Although the most common breast cancer susceptibility genes are *BRCA1* and *BRCA2*, which are also associated with the risk of developing ovarian and pancreatic cancer, advances in next-generation sequencing (NGS) analysis technology enabled the discovery of several non-*BRCA* genes responsible for breast and ovarian cancers. Studies on hereditary breast and ovarian cancer (HBOC) involve not only determining the predisposition to developing cancer, but also considering the current treatment for breast cancer, prevention of next cancer, risk diagnosis, and adoption of protective measures for relatives. We present a comprehensive review of HBOC, which will be a useful resource in the clinical setting. Many hereditary tumors, including HBOC, are syndromes characterized by the development of different types of cancer in succession. Taking advantage of knowing predisposition of susceptibility to cancer, it is important to continue and update cancer management protocols, which includes the adoption of preventive measures, countermeasures, and treatments, to accurately assess and prevent the impact of cancer on the quality of life of the next generation of patients.

## Introduction

Breast cancer is the most common cancer in women. According to the World Health Organization (WHO), the number of new cases of breast cancer in 2018 exceeded 2 million [1], accounting for 11.6% of all new cases of cancer in both men and women. The cumulative risk of developing breast cancer in the age range of 0–74 years was 9.32 in North America, 2.81–4.17 in Asia, and the highest in Australia and New Zealand at 10.16. This shows that breast cancer is common cancer affecting a large number of patients. Besides, 5–10% of all breast cancer patients are genetically predisposed to cancers. Studies on hereditary breast and ovarian cancer (HBOC) involve not only determining the predisposition to developing cancer, but also considering the current treatment for breast cancer, prevention of next cancer, risk diagnosis, and adoption of protective measures for relatives. This is a comprehensive review of HBOC, which will be a useful resource in the clinical setting.

### Hereditary breast and ovarian cancer (HBOC)

Families with a history of multiple breast or ovarian cancers approximately account for 15% of all patients with breast cancer [2], and the disease is termed familial breast cancer (FBC). FBC includes people who are genetically predisposed to cancer. According to the National Cancer Institute, HBOC is defined as “An inherited disorder in which the risk of breast cancer (especially before the age of 50 years) and ovarian cancer is higher than normal.” Most cases of HBOC syndrome are caused by certain mutations in *BRCA1* or *BRCA2.* People with HBOC syndrome may also have an increased risk of developing other types of cancer, including melanoma, pancreatic and prostate cancers. This review will discuss the current knowledge regarding hereditary breast and ovarian cancer syndrome [3].

### Human *BRCA1* and *BRCA2*

**Fig. 1:**
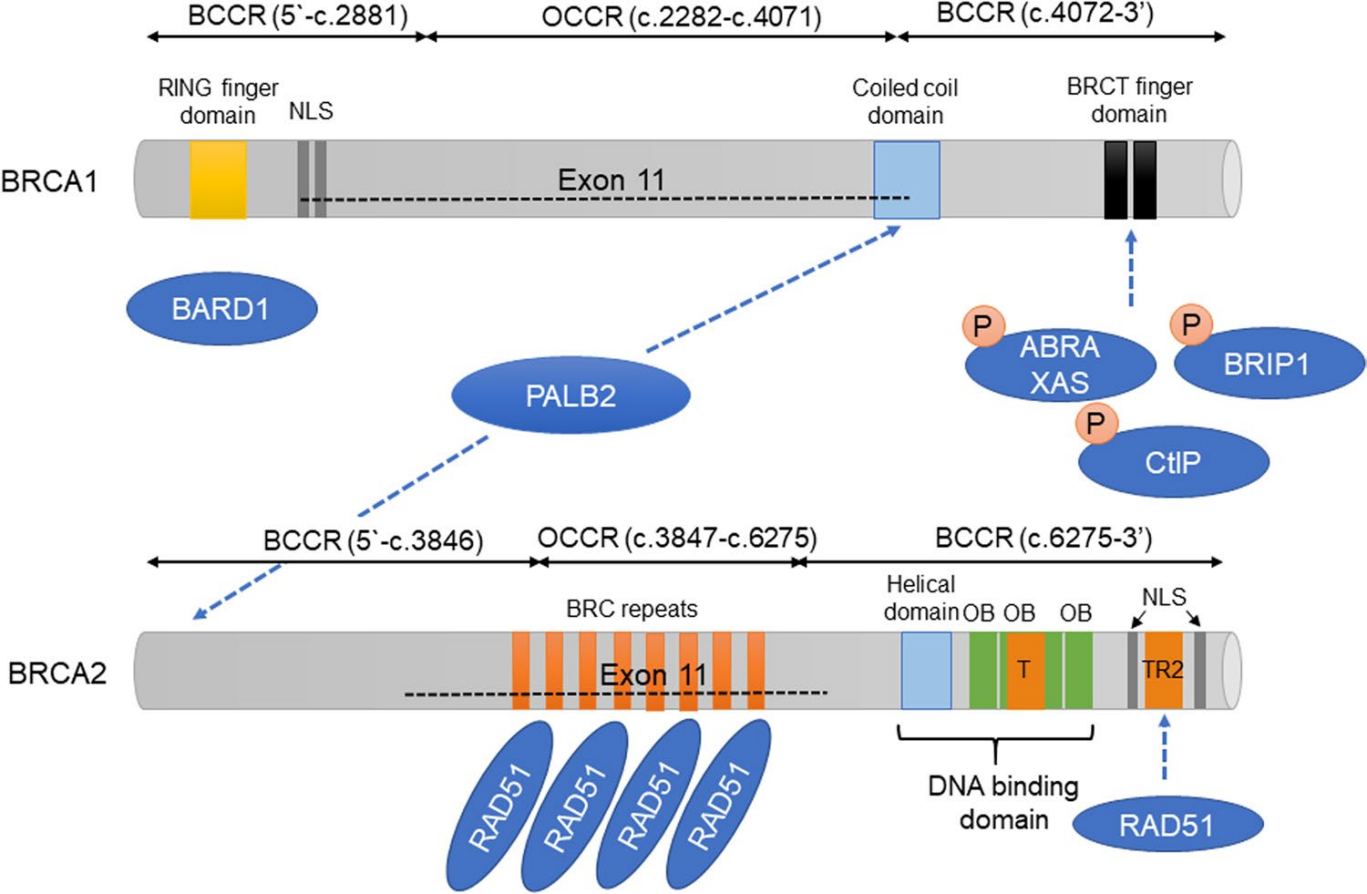
BRCA protein. BRCA1 and BRCA2 are large hub proteins that bind other molecules involved in HR. It has been reported that the phenotype of breast and ovarian cancer differs depending on the location of the mutation (BCCR, OCCR [48])

King et al. identified *BRCA1* as the cause of hereditary breast cancer via linkage analysis using genetic polymorphism markers in 23,146 young individuals belonging to families affected by breast cancer. The existence of the gene was reported in 1990 [4]. In 1994, Miki et al. successfully cloned the gene and revealed its entire structure [5]. *BRCA1* is located at 17q21 near the centromere of the long arm of chromosome 17 and has 24 exons. The protein, composed of 1863 amino acids, consists of a central N-terminal RING finger domain, a C-terminal BRCT domain, and exons 11–13 (Fig. 1). The N-terminal RING finger domain of BRCA1 binds to BRCA1-associated RING domain protein 1 (BARD1), which is structurally similar to BRCA1 and forms a RING dimeric ubiquitin ligase (E3) [6]. Moreover, the C-terminal BRCT domain binds to phosphorylated proteins [7, 8], such as BRCA1-A complex subunit (ABRAXAS) and BRCA1 interacting protein C-terminal helicase 1 (BRIP1), BTB and CNC homology 1 (BACH1), FANCJ, and C-terminal binding protein 1 (CtBP1) interacting protein [9]. The complexes containing these proteins are called BRCA1-A, -B, or -C complexes. BRCA2 binds (FNACN) to the coiled-coil domain located near the C-terminal end encoded by exon 11 via PALB2 [10].

*BRCA2* (breast cancer susceptibility gene 2) was identified in 1995 by Wooster and colleagues, by analyzing *BRCA1* mutation-negative breast cancer families, including male patients with breast cancer [11]. *BRCA2* is located on chromosome 13 (13q12-13), has 27 exons, and encodes a protein of 3418 amino acids. The N-terminus of BRCA2 contains the transcription activation domain (TAD), while the middle part is encoded by exon 11 and contains eight conserved motifs called BRC repeats, which bind to RAD51 [12]. The DNA binding domain is located at the C-terminal end of BRCA2 and consists of a helical domain, three oligonucleotide-binding (OB) folds, and a tower domain (T). Double-stranded DNA (dsDNA) and single-stranded DNA (ssDNA) promote the binding of BRCA2 with [13]. The C-terminus of BRCA2 contains two NLSs (nuclear localization signals) and one TR2 (C-terminal RAD51 binding site).

### Functions of BRCA and its role in carcinogenesis

DNA, which harbors genetic information, is constantly damaged by various internal and external factors, and is repaired via DNA single-strand break (ssDNA) repair, double-strand break (dsDNA) repair, and base mismatch repair (MMR). Depending on the type of damage, DNA is repaired via base excision repair (BER) and nucleotide repair (NER) [14]. *BRCA1/2* are caretaker tumor-suppressor genes that repair DNA double-strand breaks via homologous recombination repair (HRR) to maintain genomic stability [15]. In addition to HRR, they also control centrosome dynamics, chromosome segregation, and cytokinesis, and stabilize the genome temporally and spatially in the cell cycle. In addition to the genomic instability caused by the disruption of these functions, a hormone-dependent carcinogenic environment [16] contributes to the basic flow via which breast cells are transformed into malignant phenotypes due to accelerated activation of survival signals. In addition to their role in DNA damage repair, BRCA1 is involved in more functions than BRCA2, such as in healthy embryonic development and onset of breast and ovarian cancer [17], centrosome replication [18], regulation of spindle pole synthesis [19], heterochromatin-satellite RNA expression [20], mesenchymal metabolite synthesis [21], splicing [22], brain size [23], and transcriptional co-activation [24].

### Pathogenic variant (PV) of *BRCA*

Currently, ClinVar, which is a freely available, public archive of human genetic variants and interpretations of their significance to disease, has registered more than 2,900 variants of *BRCA1* and more than 3,400 variants of *BRCA2* as pathogenic germline mutations [25]. The 80% of pathogenic or suspected pathogenic variants generate immature stop codons, truncate the encoded protein, and reduce their expression via nonsense-mediated mRNA decay (NMD). A truncating variant [26] with missense mutations accounts for 10% of these mutations. Pathological missense mutations tend to be confined to the RING and C-terminal tandem BRCT domains of *BRCA1*, or the region spanning the OB-fold and helical domains of *BRCA2* [27]. Approximately 10% of the copy number abnormalities detected using deletion/duplication analysis varies among populations [28–31]

Genetic alterations are also observed at a high frequency in groups that are or were geographically or culturally isolated, in which one or more of the ancestors harbored the altered gene. This is often called the founder effect or founder variant [3]. Founder mutations of *BRCA1/2* have been widely reported in different regions and ethnic groups. However, genetic testing for *BRCA1/2* should include uniform sequence analysis along with deletion/duplication analysis, except for in Ashkenazi Jews. Ashkenazi Jews can undergo targeted analysis of three *BRCA1* and *BRCA2* pathogenic founder mutations; 98–99% of the PVs identified in Ashkenazi Jews are c.68_69delAG and c.5266dupC for *BRCA1*, and c.5946delT for *BRCA2* [32–34]. If any PV cannot be identified using target analysis, sequence analysis, and deletion/duplication analysis, multiple gene panel analysis should be performed. Recently, many founder mutations have been reported in Asia (Tables 1, 2).

**Table 1:** Reports of founder mutation (Caucasian)

Population	*BRCA1*	*BRCA2*	Results	References
Ashkenazi Jews	c.68_69delAG		98–99% of *BRCA1/2* mutations	[35, 36]
	c.5266dupC			
		c.5946delT		
Iceland		c.767delG	Vast majority of *BRCA1/2*	
Russia	c.5263insC		94% of *BRCA1* mutation	
Poland	c.5263insC		80% of *BRCA1/2* mutations, 91% of *BRCA1* mutations	
	c.181T>G			
	c.4034delA			
Germany	c.5263insC		38% of *BRCA1* mutations	
	c.181T>G			
	Exon17 del			
Hungary	c.5263insC	c.9098insA	80% of *BRCA1* mutations, 48% of *BRCA2* mutations	
	c.181T>G	c.5946delT		
	c.68_69delAG			
Norway	c.1556delA		68% of *BRCA1* mutations c.1556delA and c.1016insA 3% of ovarian cancer	
	c.1016insA			
	c.697delGT			
	c.3228delAG			
Finland	c.4096+3A>G	c.9117+1G>A	84% of *BRCA1/2* mutations	
	c.4327C > T	c.7480C>T		
		c.8327T>G		
		c.3376delTT		
Sweden	c.3052insC	c.6373delA	70% of *BRCA1/2* mutations in West Sweden	
Denmark	c.2475delC	c.1310del4	35% of *BRCA1/2* mutations	
	c.3319G>T	c.5946del4		
	c.5263insC			
	c.3709delT			
French	c.3481del11		52% of *BRCA1/2* mutations	
	G1570X

**Table 2:** Reports of founder mutation (Asian)

Population	Mutation in *BRCA1*	Mutation in *BRCA2*	Proportion of *BRCA1/2*	References
Southern Chinese	c.981_982delAT	c.3109C>T	23% of *BRCA1/2*	[37]
		c.7436_7805del370		
		c.9097_9098insA		
Mainland Chinese	c.981_982delAT	c.3195_3198delTAAT	5% of *BRCA1/2*	[38]
		c.5576_5579delTA		
Japanese	c.188T>A		c.188T>A variant 16% of *BRCA1/2*	[39, 40]
Koreans	c.3627insA	c. 7480C>T	10% of *BRCA1/2*	[41, 42]
	c.922_924delAGCinsT		10% of *BRCA1/2*	[42]
			10% of *BRCA1/2*	
Malaysians	c.2727insA		6% of *BRCA1*	[43]
Filipinos		c.4037delCT	13% of *BRCA1/2*	[44]
		c.4631delA	26% of *BRCA1/2*	
	c.5335delC		13% of *BRCA1/2*	[45]

### Prevalence of *BRCA* mutations

*BRCA1/2* is the most frequent cause of high penetrance among HBOCs and affects all ethnic groups and races. The frequency of *BRCA1/2* pathogenic variants in the general population, excluding Ashkenazi Jews, has been estimated to be one in 400–500 [46, 47]. The mutation frequency of the first three variants of Ashkenazi Jews: c.68_69delAG of *BRCA1*, c.5266dupC, and c.5946delT of *BRCA2* is 1 in 40 [48]. Many recent reports have determined the frequency of *BRCA1/2* mutation retention in patients with breast and ovarian cancers without selection bias, without considering family history, age of onset, and cancer subtype. Among all patients with breast cancer without selection bias, the *BRCA1/2* mutation retention rate was 4.2–6.1% (*BRCA1:* 1.45–3.7%, *BRCA2*: 2.4–3.5%) [48–52]. Among patients with ovarian cancer, the retention rate of *BRCA1/2* mutation was 8.3–14.7% (*BRCA1*: 3.4–9.9%, *BRCA2*: 4.7–5.3%) [53–56].

### Risk assessment for patients with *BRCA* mutations

**Fig. 2:**
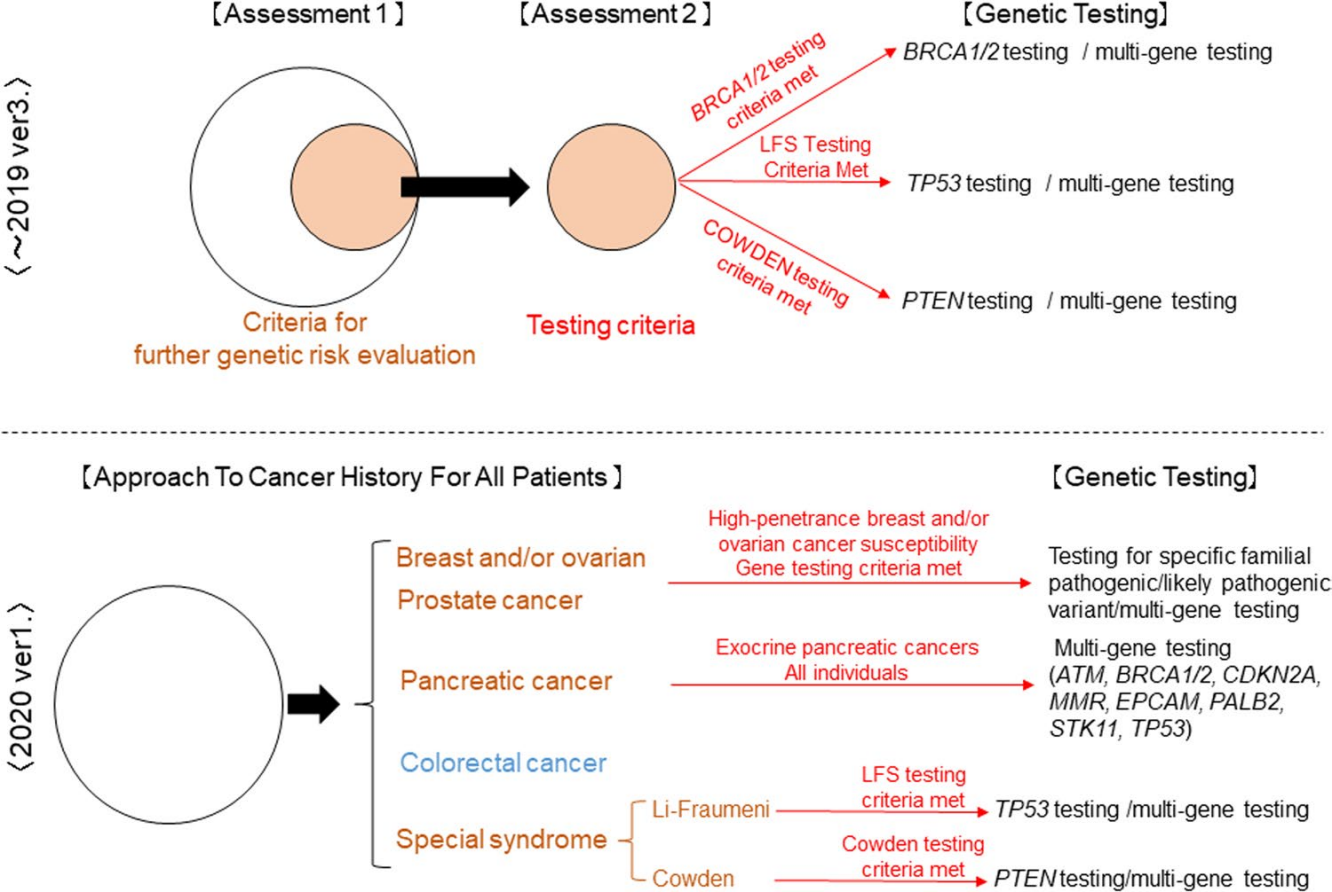
Risk assessment for HBOC. HBOC risk assessment is recommended to be comprehensive for all cancer patients instead of the traditional individual approach

As a standard for *BRCA1/2* testing, clinical judgment is often made based on medical and family history. A representative example is the National Comprehensive Cancer Network Clinical Practice Guidelines in Oncology (NCCN Guidelines) [57]. To date, the NCCN guidelines have recommended a two-stage risk assessment, 1st step: further genetic risk evaluation, and 2nd step: testing criteria, based on clinical findings of breast and ovarian cancer, as a comprehensive approach to genetic testing in subjects suspected of harboring *BRCA1/2* mutations. However, with the recent advent of PARP inhibitors, which are expected to be effective against HRR-deficient cancer, and the popularity of genetic testing that detects HBOCs other than those with *BRCA1/2* mutations, the current status of clinical practice, the 2020 version 1 called the “Approach To Cancer History” for all patients, has been revised to include recommendations for a more comprehensive approach for subjects who request genetic testing for HBOC-related genes (Fig. 2).

Many models have been developed to genetically and statistically predict *BRCA1/2* mutation retention based on past and family histories [58–61]. Although the quantified thresholds derived from these models may not be sufficient to determine whether *BRCA1* or *BRCA2* has a high probability of incorporating pathogenic variants, we cannot judge the validity of genetic testing. However, these models should be used as reference data for predicting *BRCA* mutation carriers.

### *BRCA1* and *BRCA2* are high-risk genes for breast and ovarian cancers

Studies have shown that patients with breast and ovarian cancer, including fallopian tube and primary peritoneal cancer have high risk for harboring *BRCA1/2* mutations. In addition, the risk of developing prostate cancer, pancreatic cancer, and malignant melanoma increases in the presence of these mutations. Estimates of malignant tumor risk vary with the reporting cohort and the method of risk evaluation.

**Fig. 3:**
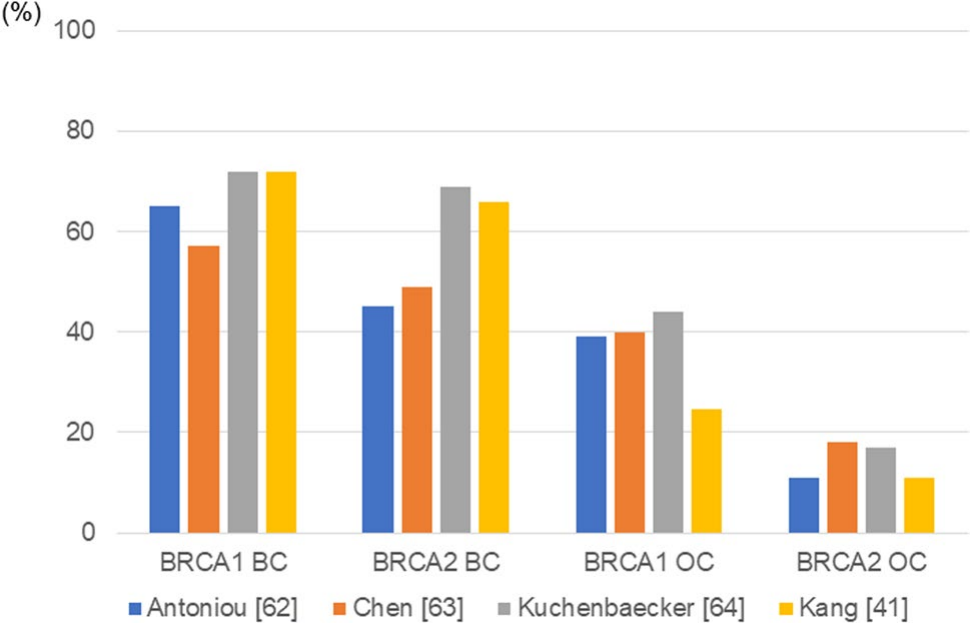
Cumulative risk of breast and ovarian cancer (women). Four studies reported the prevalence of breast and ovarian cancers in women up to the age of 70 [41, 62, 63] and 80 [64] carrying *BRCA* mutations. Two reports, [62] and [63] are meta-analysis studies, covering 22 and 10 studies, respectively, and [63] is the most extensive prospective study with 3986 *BRCA1* and 5066 *BRCA2* cancer-free mutation carriers; [41] is the Asian cohort study, involving 108 Korean *BRCA1/2* mutation carriers. Data from a large number of prospective studies are desirable for calculating the risk of developing a rare genetic disorder, as family history is prone to bias

Results from 24 studies showed that up to 70 years of age, the risk of women developing *BRCA1* mutation is 46–87%, while that for *BRCA2* mutation is 38–84%. The risk of developing ovarian cancer with a *BRCA1* mutation is 39–63%, while it is 16.5–27% for *BRCA2*; the risk for developing male breast cancer due to *BRCA1* mutation is 1.2%, while it is 8.9% for *BRCA2*; the risk of developing prostate cancer in men up to 65 years of age due to *BRCA1* mutation is 8.6%, while it is 15% for *BRCA2*; the risk of developing pancreatic cancer due to *BRCA1* mutation is 1–3%, while it is 2–7% for *BRCA2* mutation [32]. Two retrospective meta-analysis studies [62, 63] and a large prospective study [64] are shown in Fig. 3. However, since *BRCA1*/2 mutations are rare in the general population, most retrospective penetrance estimates have been derived from family-based studies, which are prone to bias, if analyses are not correctly adjusted for the ascertainment process or in case of inaccurate family history. In addition, despite the retrospective nature of our cohort study, data for the Asian population, including a report from Korea, are valuable to clinicians [41]

### *BRCA* contralateral breast cancer (CBC) risk

*BRCA1/2* mutation carriers also have a significantly increased risk of developing CBC [65, 66]. The 5-, 10-, and 15-year cumulative CBC risk of 810 female *BRCA1/2* breast cancer patients is 13.7%, 23.8%, and 36.1% for *BRCA1* and 12.0%, 18.7%, and 28.5% for *BRCA2*, respectively [67]. The overall risk of CBC in women with *BRCA1/2* mutations was 2.2%, and the annual risk for breast cancer in patients aged 40 years and younger increased to 2.8%. A prospective cohort study of 1305 *BRCA1* positive and 908 *BRCA2* positive breast cancer patients found that the risk of CBC 20 years after initial breast cancer was 40% for *BRCA1* and 26% for *BRCA2*. The younger the age of onset of the first breast cancer, the higher the risk of CBC [64].

### *BRCA* genotypes and phenotypes

The relationship between mutation sites and breast and ovarian cancer risks has been reported. In patients harboring both *BRCA1* and *BRCA2*, multiple ovarian cancer regions (OCCR) have been identified within or adjacent to exon 11 [68]. PVs in OCCR increase the proportion of ovarian to breast cancer, unlike families with PV at other sites in both genes. Similarly, a large number of breast cancer-prone regions (BCCRs) have been observed for *BRCA1/2* and are associated with a relative increase in breast cancer risk and a relative decrease in ovarian cancer risk. A prospective cohort study of *BRCA1/2* cancer-free mutation carriers [64] also reported that the relative risk of developing cancer varies with the location of the mutation (BCCR, OCCR). However, the reported hazard ratio for cancer development due to differences in the location of the mutations is almost two or less, and it is premature to use it for individual risk assessment and management. The clinical application should be considered in the future after an appropriate experimental validation.

### Radiation risks of patients with *BRCA* mutations

Radiation causes DNA damage, either directly by ionization or indirectly by radicalizing molecules, such as water, causing DNA damage, such as DNA base changes, cross-linking of DNA and proteins, and DNA single- or double-strand breaks. As these changes are known to cause cancer, radiation-induced carcinogenesis may occur in *BRCA* mutation carriers, as *BRCA1/2* is involved in double-strand break repair. Several prospective and retrospective studies have investigated the effect of medical radiation exposure on carriers of *BRCA* mutations. A retrospective cohort study, GENE-RAD-RISK, reported a relationship between medical radiation exposure, including mammography and breast cancer in 1,993 women with unaffected *BRCA1/2* mutations. Patients who received mammography before age 30 had an increased risk of breast cancer compared to those who were not exposed (hazard ratio = 1.43) [69]. However, questionnaire surveys suggest that this study may have recall bias. Subsequently, a prospective study investigating the effects of mammography on 2346 women with *BRCA* mutations, considered the 5-year cumulative breast cancer incidence and reported no significant difference in the risk of developing breast cancer relative to the mammography history and mammography initial age [70]. Although the relationship between carriers of *BRCA* mutations and the development of radiation-induced breast cancer is not yet clear, after considering the age of breast cancer occurrence and the benefits of surveillance using mammography, many guidelines recommend MMG screening from the age of 25.

## Characteristics of *BRCA*-related breast and ovarian cancer

### Pathology of breast and ovarian cancer

The Western Consortium of Investigators of Modifiers of BRCA1/2: the CIMBA reports on the pathological findings of *BRCA1/2* breast cancer as follows [71, 72]: *BRCA1*-related breast cancer has the following features: (1) histopathological image of medullary carcinoma, which develops in a globular manner in peripheral tissues, (2) high histological nuclear grade, (3) a high proportion of negative for the expression of both estrogen and progesterone receptors and HER2 overexpression. The histology of the *BRCA2*-mutated breast cancer tissue is almost similar to those without *BRCA* mutations, and the histological nuclear grade is generally high (Table 3). In addition, high-grade serous adenocarcinoma has been reported as a pathological feature of *BRCA1/2* ovarian cancer [71, 73]. Both *BRCA1/2*-related breast and ovarian cancers are typically highly aggressive (Table 3).

**Table 3:** Pathological features of *BRCA1/2*-related breast and ovarian cancer

	*BRCA1*		*BRCA2*	
Breast cancer	Mavaddat [71]	Kuchenbaecker [72]	Mavaddat [71]	Kuchenbaecker [72]
Histology				
Invasive ductal	80%	82%	83%	79%
Invasive lobular	2.20%	2%	8.40%	8%
Medullary	9.40%	6%	2.20%	2%
Other	8.60%	10%	6.40%	11%
Grade				
Grade 1	3%	3%	7%	7%
Grade 2	20%	18%	43%	42%
Grade 3	77%	79%	50%	51%
ER, PgR, HER2, TN				
ER-positive	22%	24%	77%	77%
PgR-positive	21%	21%	64%	65%
HER2-positive	10%	9%	13%	13%
Non-TN	31%	31%	84%	85%
Ovarian cancer	Mavaddat [71]	McLaughlin [73]	Mavaddat [71]	McLaughlin [73]
Histology				
Serous	66%	73.60%	70%	73%
Mucinous	1%	0%	1%	0%
Endometrioid	12%	14.70%	12%	8.90%
Clear cell	1%	0.80%	3%	2.30%
Other	20%	10.90%	13%	15.70%
Grade				
Grade 1	3%	1.60%	6%	0%
Grade 2	20%	15.50%	21%	21.40%
Grade 3	77%	56.60%	73%	52.80%
Unknown	–	26%		25.80%

Mavaddat: (2011 3,797 BRCA1 mutationcarriers and 2,392 BRCA2 mutation carriers diagnosed with invasive breast cancer., 838 BRCA1 mutation carriers and 281 BRCA2 mutation carriers who had been diagnosed with ovarian cancer Kuchenbaecker(2014 Breast tumor characteristics of 7,797 affected BRCA1 mutation carriers and 4,330 affected BRCA2 mutation carriers) McLaughlin(2013: 129 BRCA1 mutation carriers and 89 BRCA2 mutation cariers diagnosed with ovarian cancer)

### Prognosis of *BRCA* cancer

The prognosis of breast cancer in *BRCA1/2* mutation carriers has been reported extensively, and the results of large-scale cohorts and meta-analyses in this regard are as follows. Pooled analysis of 16 studies, including 1325 with *BRCA1/2* mutation and 8855 with no mutation regarding the overall survival of patients with breast cancer harboring *BRCA1/2* mutations, revealed a correlation between the presence of *BRCA1/2* mutation and overall survival. No correlation was observed in terms of hazard ratio (hazard ratio = 1.06, *p* = 0.61) [74]. In addition, the results of a 127 multi-center prospective cohort study in the UK involving 2733 women with breast cancer under the age of 40 years, including 388 *BRCA1/2* mutation carriers, showed no relationship between the presence of *BRCA1/2* mutations, and 2-, 5- and 10-year overall survival. In a cohort consisting of 588 patients with triple-negative breast cancer, the *BRCA1/2* mutation carriers showed significantly higher 2-year overall survival rates (hazard ratio = 0.59, *p* = 0.047); however, no difference at 5- and 10-years was reported [75]. This slight early survival advantage might be linked to greater sensitivity of BRCA-mutant breast cancers to chemotherapy. A meta-analysis of 33 studies of patients with ovarian cancer showed that overall survival (hazard ratio = 0.75, *p* < 0.001) and progression-free survival (hazard ratio = 0.80) in the *BRCA1/2* mutation cohort (*p* = 0.039) were significantly extended compared to that of patients with no *BRCA* mutation. *BRCA1* mutation alone improved overall survival (hazard ratio = 0.75), although no significant difference in progression-free survival was observed; besides, no significant difference in overall or progression-free survival was observed for patients carrying *BRCA2* mutation alone. In a large study involving 626 patients with ovarian cancer without selection bias, patients with 218 *BRCA1/2* mutations had better 3-year short-term survival than the mutation-free group. However, reports show that this effect on survival is short-term and that long-term survival beyond 10 years does not improve [73].

### Locus-specific loss of heterozygosity (LOH)

A few reports suggested a correlation between prognosis and the presence or absence of LOH in cancers of germline *BRCA1/2* mutation carriers. Of 160 cancers with *BRCA1/2* mutations, LOH was detected in 90% cases of *BRCA1* positivity and 54% cases of *BRCA2* positivity in breast cancer, and in 93% cases of *BRCA1* positivity and 84% cases of *BRCA2* positivity in ovarian cancer. In patients with breast cancer, the overall survival rate was better in the *BRCA1/2*-positive group than in the negative group; however, overall survival did not differ significantly with the presence or absence of LOH in cancer. The overall survival rate of patients with *BRCA*-positive cancers was significantly better, with that of *BRCA1/2*-positive groups without LOH being as low as that of *BRCA*-negative groups [76]. The presence or absence of LOH in tumors should be considered as a new stratification factor when predicting the effects of drugs, such as PARP inhibitors.

## Non-*BRCA* genes related to HBOC

### HBOC-related genes and multi-gene panel (MGP) testing

**Fig. 4:**
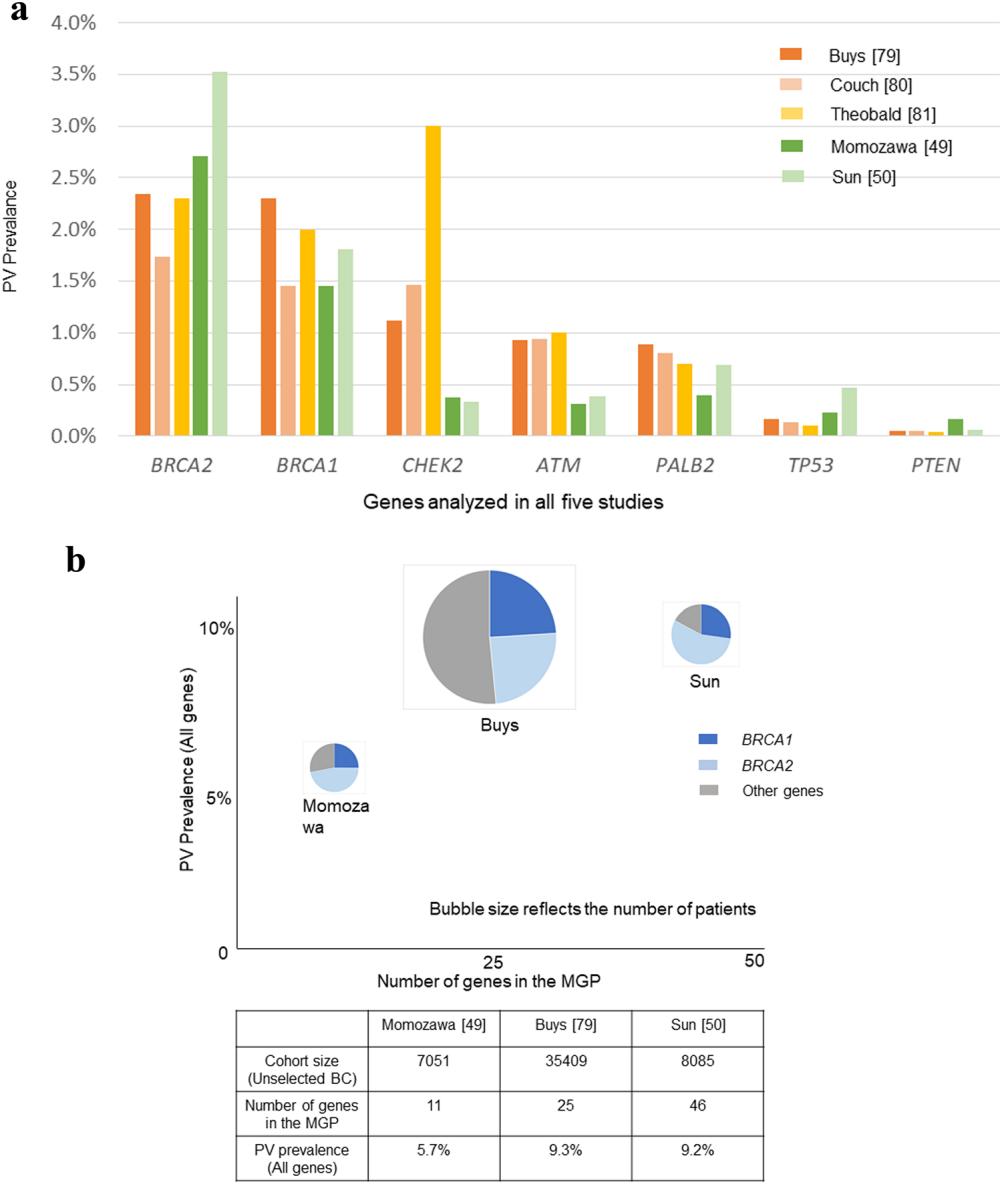
**a** Results of MGP testing for unselected patients with breast cancer (*ATM, BRCA1, BRCA2, CHEK2, PALB2, PTEN, TP53*). Genetic prevalence is suggested to differ by cohort background and ethnicity. **b** PV prevalence (all genes). *BRCA2* mutation prevalence is still high in Asian cohorts, even as the number of genes examined increases

A review of the causative genes of FBC showed that only 25% of cases retained the *BRCA1/2* mutations, whereas four high-susceptibility genes, other than *BRCA1/2*, namely *CDH1, PTEN, STK11,* and *TP53*, were detected in 5% of patients. Another 5% of cases reported medium penetrance genes (RR: 2–4), and 14% low penetrance genes (RR < 2), whereas the causative gene was unknown in 51% cases [77]. Nearly all known HBOC susceptibility genes encode tumor suppressors that participate in genome stability pathways, in particular HRR, and to some extent mismatch repair (MMR), as well as interstrand DNA cross-link repair via the Fanconi anemia pathway [78]. Advances in next-generation sequencing (NGS) analysis technology enabled cost-effective and increasingly efficient genetic testing. Hence, from the era of investigating single genes one at a time, we have now advanced to MGP testing, which allows to assess many candidate genes simultaneously. Various types of MGP testing for HBOC are currently available. The results of gene mutation detection rates using MGP testing in patients with over 7000 breast cancer without selection bias are shown in Fig. 4a (Momozawa et al. [64], Sun et al. [65], Buys et al. [79], Couch et al. [80], Theobald et al. [81]). References [79–81] report the results of a testing company mainly targeting Caucasians, and references [64, 65] report cohort studies focusing on Asians. In the Caucasian-centered cohort, *BRCA1* and *BRCA2* showed similar PV prevalence; however, in Asian cohorts, the prevalence of *BRCA2* tended to be high, whereas that of *CHEK2/ATM* tended to be low. In addition, genes with PV were analyzed between the three of five studies, which analyzed genes using only one-panel testing in their cohort (Fig. 4b). *BRCA2* mutation carriers showed high penetrance in Asian cohorts, even when more genes were considered. The following points should be noted when performing MGP testing. According to a review report summarizing the results of 23 MGP testing, the variant of uncertain significance (VUS) detection rate was as high as 0.6–88%. It is necessary to pay attention to the economic and mental burden of carrying mutations for which medical management is unknown [82]. The utility of the MGP test is not only to detect a high prevalence of PV, but also to expand the possibility of finding genetic diseases that were not expected from information on family and individual medical histories, and to take new measures. Identification of pathogenic variants in genes associated with cancer can open new therapeutic avenues.

### Clinical features of high penetrance genes in breast cancer

The clinicopathological findings regarding the high penetrance genes involved in breast cancer, including *BRCA1/2*, are shown in Table 4 [83–87]. The mean age at which cancer develops is lower in patients with *TP53* and *PTEN* mutations than in those with *BRCA1/2* mutations, and therefore, it is necessary to pay attention to the age at which surveillance begins. Although large-scale evidence is still scarce for medium- to low-risk genes, limited studies on *PALB2*, *ATM*, *CHEK2,* etc., are being reported in fast succession, and it is expected that the clinicopathological features and appropriate medical management will be further clarified in the future by accumulating data offered by MGP.

**Table 4:** Clinicopathological features of high penetrance HBOC genes

	*BRCA1 BRCA2*	*TP53* (Li-Fraumeni syndrome)	*PTEN* (Cowden syndrome)	*CDH1* (Hereditary diffuse gastric cancer)	*STK11* (Peutz-Jeghers syndrome)
Component tumor	Breast, Ovary, Prostate, Pancreatic	Adrenal Gland	Breast, Endometrioid	Gastric	Gastrointestinal
		Breast (premenopausal), brain	Colorectal	Breast	Breast, Ovary, Endometrioid
		Soft tissue/bone	Thyroid	Colorectal	
		Sarcoma	Renal		
Breast cancer penetrance (women)	*BRCA1*: 71% (~ 80 years)	54% (~ 70 years)	67–85%	39–52%	45–50%
	*BRCA2*: 69% (~ 80 years)				
Age of breast cancer onset (average)	BRCA1: 44 years	33 years	38 ~46 years	53 year	Insufficient data
	*BRCA2*: 48 years				
PV prevalence 35,000 breast cancer cohort [79]	*BRCA1*: 2.30%	0.17% 4–8% of *BRCA*-negative breast cancers under 30 years of age are *TP53* mutation-positive	< 0.1%	< 0.1%	< 0.1%
	*BRCA2*: 2.34%				
Pathological feature of breast cancer	*BRCA1*: TN, 77%; NG2 or 3, 97%	*HER2*+ : 66% NG2 or 3: 86%	Insufficient data	Invasive lobular carcinoma	Insufficient data
	*BRCA2*: Luminal, 77%; NG2 or 3, 93%				
References	[32, 57, 70]	[32, 57, 84, 85]	[32, 57, 85]	[32, 57, 86]	[32, 57, 87]

### Treatment for current breast cancer

#### Surgery

It is generally recognized that breast-conserving surgery (BCT) is an alternative to mastectomy for early breast cancer. However, the relative contraindications for BCT with breast irradiation in the NCCN guidelines include "women with known or suspected genetic predisposition to breast cancer" [88]. The possible reasons include increased risk of ipsilateral breast recurrence or CBC in women with HR-related or radiosensitive germline mutations, such as Li-Fraumeni syndrome, complications of breast reconstruction after breast irradiation, and consideration of prophylactic bilateral mastectomy (BRRM). The risk of developing ipsilateral breast recurrence and CBC survival after BCT in *BRCA1/2* mutation carriers has long been discussed. A recent review examined six retrospective studies on BCT toxicities in *BRCA* mutation carriers and non-carriers, six retrospective studies on BCT and mastectomy in *BRCA* mutation carriers, one review of 11 retrospective studies on BCT of IBTR, CBC, distant recurrence, and overall survival, and one meta-analysis report [89]. Although it is unclear whether the risk of long-term IBTR, including new primary IBTR, increases after BCT in *BRCA* mutation carriers and non-carriers, no significant differences in BCT toxicities, distant recurrence and overall survival between BCT and mastectomy in *BRCA* mutation carriers were reported. To date, no randomized controlled trials of BCT with radiation therapy for breast cancer with *BRCA* mutations are available. The available information is drawn from retrospective studies, case reports, or reviews.

Because of the risk of developing new cancers in the long term, mastectomy is not recommended unless *BRCA* alterations are detected preoperatively or in case the patient strongly desires breast-conserving surgery.

### Systematic therapy

Abnormality in the *BRCA* gene impairs the DNA repair pathway, resulting in the accumulation of damaged DNA. Reportedly, patients with *BRCA* mutations are highly sensitive to drugs that cause DNA damage, such as platinum doublet and PARP (poly (ADP-ribose) polymerase) inhibitors.

### Platinum doublet

In a Phase III TNT study, to compare the effectiveness of carboplatin and docetaxel in TNBC or germline *BRCA1/2* mutation-positive breast cancer with metastatic recurrence, both overall response rate (ORR) and progression-free survival (PFS) were significantly improved in the carboplatin-treated group (ORR: 68% vs. 33.3%, median PFS: 6.8 months vs. 4.4 months). However, no difference was observed in overall survival (OS) [90]. On the other hand, in neoadjuvant chemotherapy, the subgroup analysis of TNBC in the GeparSixto study showed that even if paclitaxel, non-pegylated liposomal doxorubicin, and bevacizumab were combined with carboplatin among germline *BRCA* mutation-positive patients, the pCR rate did not change. It has been suggested that the effect of carboplatin may not have been observed due to the DNA repair fraud effect of doxorubicin [91].

### PARP inhibitor

Tumors with HRR abnormalities, such as *BRCA-*mutant cancers, are known to be sensitive to platinum products that cause interstrand cross-linking (ICL). Although various drugs have been developed, the search for biomarkers that can predict their effectiveness is underway. PARP is an enzyme that participates in the repair of single-strand breaks in DNA via the BER pathway. If DNA single-strand break repair is impaired, the single-strand break is converted to a double-strand break and repaired via the double-strand break repair machinery. However, in cells with impaired HRR function, such as *BRCA*-mutant cancer, repair via non-homologous end joining (NHEJ) or microhomology-mediated end joining (MMEJ), may occur. NHEJ and MMEJ are highly error-prone repair mechanisms, and cells repaired via these mechanisms undergo complex genomic rearrangements and cell death. This series of events is called synthetic lethality [92]. Two Phase III studies tested PARP inhibitors for metastatic recurrent HER2-negative germline *BRCA*-positive breast cancer, OlympiAD, comparing Olaparib alone with standard physicians’ choice of chemotherapy, showed a significant prolongation of PFS with a median of 7.0 months versus 4.2 months [93]; another trial, EMBRACA, compared talazoparib monotherapy with standard physicians’ choice of chemotherapy, and also showed significantly prolonged PFS with a median of 8.6 months versus 5.6 months [94]. These two drugs are drugs approved in many countries, including by the FDA. At present, clinical trials with germline *BRCA* mutation-positive patients using PARP inhibitors and other agents, including platinum doublet, ATR inhibitors, anti PD-L1, etc., are in progress. On the other hand, a Phase III study (OlympiA study) is underway to verify the usefulness of additional Olaparib in patients with high-risk recurrence who are germline *BRCA* mutation-positive after neoadjuvant or adjuvant chemotherapy.

Reportedly, PARP inhibitors may be effective for tumors that share features of BRCA-mutant tumors—that is, those with ‘BRCAness’ [95], including BRCA cancers. At present, various tools for predicting HRD have been developed, but it is unclear which HRR assay has the highest sensitivity for predicting the effect of a drug. The results of clinical trials are awaited (Table 5) [96–98].

**Table 5:** Current HRR assays

	BRCAness®	myChoice® HRD	HRDetect
Sample	FFPE	FFPE	FF
Method	Tumor samples were measured using the MLPA method for 34 copy number abnormalities that were significantly observed in g*BRCA1*-positive tumors, and scored using PAM statistics	Analysis of BRCA1 / 2 tumor samples and analysis of genomic instability (HRD score)	Perform WGS on tumor samples and calculate 6 parameters of signature (3, 8), rearrangement signature (3, 5), deletions with MH, and HRD using logistic regression analysis
Purpose	Decision on the treatment plan for TNBC	PARP inhibitor effect prediction	Measures loss of *BRCA1 / 2* function in tumor
References	Lips [96]	Timms [97]	Davies [98]

### Cancer prevention

Knowledge regarding one’s predisposition to germline cancer enables the adoption of appropriate measures. Cancer prevention includes risk-reducing surgery and chemoprevention. Preventive care should be decided upon consultation with genetic counseling if desired. Currently, among the genes related to hereditary breast cancer, only *BRCA1/2* genes can be considered to have evidence of the medical utility of preventive care.

### Risk-reducing surgery

#### Risk-reducing mastectomy (RRM)

Contralateral breast risk-reduction resection can be performed after the onset of unilateral breast cancer, and bilateral breast risk-reduction resection can be performed for those without breast cancer. Recently, meta-analyses of breast cancer risk and mortality after bilateral and contralateral prophylactic mastectomy have been reported. In total, there are 2,555 cases for bilateral breast risk-reduction resection and 1,672 cases for contralateral resection. Breast cancer-specific risk after prophylactic mastectomy was significantly reduced to relative risk = 0.11 and 0.072 for bilateral and contralateral resections, respectively. Overall mortality did not differ significantly after bilateral prophylactic mastectomy but was significant after contralateral prophylactic mastectomy. Breast cancer-specific mortality did not differ significantly after bilateral (hazard ratio = 0.226) and contralateral (hazard ratio = 0.512) prophylactic mastectomy. However, in one of the studies, breast cancer-specific mortality after contralateral prophylactic mastectomy showed a significant difference when analyzed after extending the follow-up period from 10 to 20 years (hazard ratio = 0.52) [99].

#### Risk-reducing salpingo-oophorectomy (RRSO)

RRSO has been used successfully. A meta-analysis of 2,871 cases in three studies showed a significant reduction in the risk of ovarian cancer, with a hazard ratio of 0.21 [100], and a meta-analysis of three other prospective studies involving 9192 cases showed a decrease in overall mortality with a hazard ratio of 0.32. Overall mortality was significantly reduced in both subgroup analyses for patients with a history of *BRCA1-* and *BRCA2*-mutated breast cancer and no history of breast cancer. In addition, incidences of RRSO and breast cancer in women lacking *BRCA1/2* mutation have been reported [101]. Analysis of all *BRCA1/2* cases showed no reduction in the risk of developing breast cancer in the RRSO-treated group, although the *BRCA2* mutation-bearing group showed an 83% reduction in risk at < 50 years of age. RRSO was similarly effective in preventing breast cancer (hazard ratio = 0.10, *p* = 0.03) for women under 50 years of age who could be persuaded for analyzing their estrogen receptor positivity. *BRCA1* has a large risk-reduction effect on ovarian cancer, whereas *BRCA2* has a risk-reduction effect on breast cancer after RRSO. Thus, RRSO should be performed based on the above information. We expect that in future genetic counseling will offer more reliable predictions of the quality of life for patients carrying *BRCA1/2* mutations.

## Conclusion

Many hereditary tumors, including hereditary breast cancers, are syndromes characterized by the development of different types of cancer in succession. Taking advantage of knowing the predisposition of susceptibility to cancer, it is vital to continue management, which includes the adoption of preventive measures, countermeasures, and treatments, and assessment of the impact on the next generation of patients.
